# Supplementary material for: Performance evaluation of automotive product development team members based on a PLM system: A case study of M Automotive Products Company
Source: PLoS One. 2021 Aug 27;16(8):e0255300. doi: 10.1371/journal.pone.0255300 (PMC8396725; doi:10.1371/journal.pone.0255300)
Supplement: Attachment — Submitted filename: Response to Reviewers.docx [file pone.0255300.s002.docx]

Response to Reviewers

Dear Editors and Reviewers:

Thank you for your letter and for the reviewers’ comments concerning our manuscript entitled “Performance evaluation of automotive product development team members based on PLM system: A case study of M Automotive Products Company” (ID: PONE-D-21-01779). Those comments are all valuable and very helpful for revising and improving our paper, as well as the important guiding significance to our researches. We have studied comments carefully and have made correction which we hope meet with approval. Revised portion are marked in red in the paper. The main corrections in the paper and the responds to the reviewer’s comments are as flowing:

Responds to the reviewer’s comments:

Reviewer #1:

1. Response to comment: Highlight the contribution of the study

Response: Considering the Reviewer’s suggestion, we focused on the research value of this paper from two aspects of social value and academic value in the 1. Introduction part.

2. Response to comment: Highlight more the advantage of the system and the limitation of the study

Response: In 5. Conclusion, we summarize four advantages of PLM system and three disadvantages of this research.

3. Response to comment: Cite more recent similar articles

Response: In order to better demonstrate the superiority of the model and system and show more theoretical support, this paper quotes 18 new literatures related to project management, system development and performance evaluation.

Special thanks to you for your good comments.

Reviewer #2:

1. Response to comment: Improve the introduction part and Result & discussion part

Response: We have re-written this part according to the Reviewer’s suggestion. In the 1.Introduction part, we state the research methods and ideas of this paper in detail. In the 3. Method part, we elaborate the theory basis and model of PLM system development. In the 5. Conclusion part, we emphasize the advantages of PLM system and the research defects of this paper.

2. Response to comment: add some citation in introduction part and Result & discussion

Response: In order to better demonstrate the superiority of the model and system and show more theoretical support, this paper quotes 18 new literatures related to project management, system development and performance evaluation.

Special thanks to you for your good comments.

We tried our best to improve the manuscript and made some changes in the manuscript. These changes will not influence the content and framework of the paper. And here we did not list the changes but marked in red in revised paper.

We appreciate for Editors/Reviewers’ warm work earnestly, and hope that the correction will meet with approval.

Once again, thank you very much for your comments and suggestions.
